# Diagnosis of the Different Aspects of Fat in the Liver

**DOI:** 10.5334/jbr-btr.1050

**Published:** 2016-02-29

**Authors:** Christophe Aubé, Jérôme Lebigot, Victoire Cartier

**Affiliations:** 1 CHU Angers, FR

Liver fat infiltration usually appears as a diffuse abnormality that modifies the signal of the liver homogeneously. But this overload can be heterogeneous in different circumstances and in different ways: heterogeneous and focal steatosis and fatty focal sparing. It is important to know these aspects in order to not misdiagnose these abnormalities. The segmental repartition of the steatosis is easily explained by the different contribution to the portal flow that is both mesenteric or splenic. As portal flow is laminar and helicoidal, it could result in an incomplete mixing of splenic and mesenteric blood in the intraparen chymal liver vascularisation and therefore a preferential uptake of lipids coming from mesenteric blood flow in an area of the liver.

Heterogeneous and focal fatty depositions are partially explained by vascular theories. An excess of insulin blood supply (due to an anatomical variant of pancreatic blood vessels) is the most accepted theory, but there are probably other explanations, such as ischemic mechanisms, that take a role in these abnormalities. These focal steatoses have typical semiology: Their signal drops on opposition phase T1 EG sequences. They are geographic margins; they produce no mass effect and vessels are not distorted inside the lesion. The enhancement is homogeneous, and the enhancement ratio between fatty and non-fatty liver is preserved on the different vascular phases. Finally, there is typical distribution in the liver (posterior part of the segment IV, gallbladder fosse, falciform ligament, and subcapsular region). The last type of focal steatosis is nodular steatosis, which is a rare entity, but it is really difficult to differentiate a tumour (benign or malign) from fatty content. In this situation, if there is oncologic context or in case of doubt, a biopsy should be performed.

The second way of heterogeneous repartition of the steatosis is the heterogeneous and focal fatty sparing. This lesion is, at present, more frequent than focal steatosis. For this type of lesion, the explanation is quite clear. Anatomical vascular variants lead to a non-portal blood contribution. This is the theory of the third supply. In territory with systemic blood supply rather than portal blood supply, the uptake of lipids is low and there is no steatosis. In opposition phase T1 EG sequence, the signal of the liver is dropped except the signal of the sparing area. The semiology regarding homogeneity, vessels, and enhancement is similar to that of the focal steatosis. Preferential locations are also the same. Nodular fatty sparing is possible. In this situation, such as in the case of nodular steatosis, it is important to eliminate a real liver tumour in a fatty liver. Thus all sequences of the MRI should be carefully analysed to avoid a misdiagnosis, and in case of doubt, a biopsy should be performed.

MRI is the key examination for the diagnosis of focal fatty liver or focal fatty sparing, but contrast enhanced ultrasound (CEUS) could be really useful. The enhancement of focal fatty liver or sparing is similar to the surrounding parenchyma. This is a strong argument to make a difference with real tumours.

Focal fatty liver and, even more, focal fatty sparing are frequent lesions. They could be in the majority of cases easily diagnosed using CEUS and MRI. But in case of doubt, especially in case of nodular lesion, a biopsy should be performed.

## Competing Interests

The authors declare that they have no competing interests.

